# Changes in Antioxidant Properties and Phenolics in Sweet Potatoes (*Ipomoea batatas* L.) Due to Heat Treatments

**DOI:** 10.3390/molecules27061884

**Published:** 2022-03-14

**Authors:** Hana Franková, Janette Musilová, Július Árvay, Marek Šnirc, Ivona Jančo, Judita Lidiková, Alena Vollmannová

**Affiliations:** Institute of Food Sciences, Faculty of Biotechnology and Food Sciences, Slovak University of Agriculture in Nitra, Tr. A. Hlinku 2, 949 76 Nitra, Slovakia; janette.musilova@uniag.sk (J.M.); julius.arvay@uniag.sk (J.Á.); marek.snirc@uniag.sk (M.Š.); xjanco@uniag.sk (I.J.); judita.lidikova@uniag.sk (J.L.); alena.vollmannova@uniag.sk (A.V.)

**Keywords:** sweet potato, heat treatment, chlorogenic acids, polyphenols, antioxidant activity

## Abstract

Processing is one of the most crucial factors affecting polyphenol content in foods. Therefore, the study is aimed at the evaluation of heat treatment effects (microwaving, steaming, baking, and boiling) on the content of chlorogenic acids, total polyphenols, and antioxidant activity of three varieties of sweet potato with different flesh colors (Beauregard—orange-fleshed, O’Henry—white-fleshed, 414-purple—purple-fleshed). According to high performance liquid chromatography analysis, chlorogenic acid was the predominant chlorogenic acid in sweet potatoes. Obtained results also suggested the purple-fleshed variety (414-purple) had significantly (*p* < 0.05) higher total polyphenol content and thus the highest antioxidant activity. Heat treatment positively influenced the chlorogenic acid content, total polyphenols, and antioxidant activity of sweet potatoes. Among the used methods, steaming had the greatest effect on the chlorogenic acids and total polyphenols, while microwaved samples showed the highest antioxidant activity (DPPH). The content of chlorogenic acids and total polyphenols decreased in the order of steaming > baking > microwaving > boiling > raw. However, the individual varieties differed not only in the flesh color but also in the reaction to the used heat treatment methods. Spearman’s correlation coefficient showed a strong correlation between chlorogenic acid and antioxidant activity.

## 1. Introduction

The sweet potato (*Ipomoea batatas* L.) is a dicotyledonous viny plant and the only economically important member of the Convolvulaceae. It originates in Central and South America and is grown mainly in tropical and warm temperate climatic areas, mainly due to its tuberous roots [1,2], but is currently an important crop grown in more than 100 countries all over the world [3]. The cultivation of the sweet potato in Europe began in the 16th century and later spread to Asia [4]. In many countries of Asia and Africa, sweet potato is an important agricultural crop [5,6], which is reflected in their high production compared to other countries worldwide.

The aboveground parts of the sweet potato (stems and leaves) are not generally used for the human diet, although in some countries, they are used as feed for livestock [7]. However, especially in Asia and Africa, the young shoots and leaves of sweet potatoes are sometimes eaten as greens [5,6,7]. The advantage of sweet potato leaves is the possibility of harvesting several times a year, and their yields are much higher than the yields of green leafy vegetables [8].

Sweet potato tubers, as well as their leaves, have a high nutritional value. In general, sweet potato is a rich source of carbohydrates, fiber, minerals, and other nutrients [9]. They are particularly rich in starch [10,11]. Compared to potatoes (*Solanum tuberosum* L.), sweet potatoes have a much better nutritional potential for humans. They have a higher energy value and level of vitamin C. Sweet potato tubers are also a richer source of minerals and vitamins than potatoes [2]. Minerals in sweet potatoes are represented mainly by K, P, Ca, and Mg. They also contain minor amounts of Na and other minerals such as Fe, Zn, etc. [1,12]. 

Consumer demand for healthy foods is growing worldwide, so the nutritional value and content of biologically active substances in sweet potatoes have been attracting the attention of researchers for several years [3]. Bioactive compounds are present in food, especially in fruits, vegetables, and whole grains, and provide health benefits beyond primary nutritional value [13]. Epidemiological studies have suggested a positive effect of consumption of foods rich in bioactive substances with antioxidant activity, especially phenolic compounds, on human health. They may reduce the risk of many diseases such as cancer, heart disease, stroke, diabetes, Alzheimer’s disease, and others [14,15]. 

Sweet potatoes are an excellent root vegetable that have a high nutritional value. They are a rich source of phytochemical compounds, which have always been an important source of several clinically useful biomolecules, such as phenolic compounds, carotenoids, and anthocyanins [16]. These substances contribute to various health benefits of sweet potatoes, including antimutagenic, antioxidant, antimicrobial, anticarcinogenic, hepatoprotective, cardioprotective, and anti-inflammatory properties [3,4,15,17,18]. Polyphenols in sweet potatoes are represented by two major groups, namely phenolic acids and flavonoids [19], which are also the most important groups of phenolic compounds in food [20]. Phenolic acids are the main phenolic compounds in sweet potatoes [21]. They are characterized as derivatives of benzoic and cinnamic acid and occur naturally in foods of plant origin, mostly in bound form as esters or glycosides [22,23]. The phenolic compounds in sweet potatoes have been found to inhibit leukemia and the growth of cancer cells in the stomach and colon, and to help treat diabetes. The content of phenols, anthocyanins, and carotenoids in sweet potatoes is related to their antioxidant activity [24].

Sweet potatoes are a good source of polyphenols and are commonly cooked in the same way as other vegetables before consumption. Sweet potato is usually cooked by baking, boiling, microwaving, steaming, or frying. These cooking methods would cause many changes in the physical characteristics and change the chemical composition of sweet potatoes [25,26]. Conditions such as time, temperature, and heat treatment method are determinants that affect the antioxidant activity of crops [27,28]. The research is focused on the determination of antioxidant activity, total polyphenol content, and selected chlorogenic acids in different varieties of sweet potatoes, as well as on the evaluation of the effect of various heat treatments commonly used for sweet potatoes on the total antioxidant activity and content of selected bioactive substances in sweet potatoes.

## 2. Materials and Methods 

### 2.1. Plant Material

Three varieties of sweet potato with different flesh and peel color—Beauregard (orange), O’Henry (white), and 414-purple (purple)—were provided by Zelfer s.r.o. (Šoporňa, Slovakia). Sweet potato varieties were grown in the cadastral area of Šoporňa (Slovakia) and harvested in September 2020. Overall, about 2 kg of fresh sweet potatoes of each variety were used for heat treatment and subsequent preparation of extracts.

### 2.2. Preparation of Samples and Processing

Raw and processed sweet potato samples were used for the analysis of the total polyphenol content and antioxidant activity. The sweet potato tubers were thoroughly washed with distilled water, and peeled tubers were washed repeatedly with distilled water. The sweet potatoes thus prepared were cut into slices. The pieces approximately 3 mm wide were used for processing.

The sweet potato tubers were prepared by microwaving, steaming, baking, and boiling. The sweet potato slices were microwaved at 800 W for 5 min. For the baking, the slices were baked in preheated oven at 200 °C for 15 min. For the steaming method, the slices of each sweet potato variety were placed into a steamer and steamed at 97 ± 2 °C for 15 min. For the boiling method, the slices of sweet potatoes tubers of each variety were covered with distilled water in the stainless-steel cook pot and boiled at 100 °C for 20 min. After each heat treatment, the processed sweet potatoes were cooled and then used for the preparation of extracts.

### 2.3. Preparation of Extracts

Raw and heat-treated sweet potato samples were homogenized, and 25 g of each sample was poured with 80% methanol (50 mL) and extracted by a horizontal shaker (Heidolph Promax 1020, Heidolph Instruments GmbH, Schwabach, Germany) at room temperature for 12 h. Samples thus prepared were filtrated through Muktell No. 392 paper (Munktell & Filtrac GmbH, Bärenstein, Germany) and stored in centrifuge tubes at 4 °C in the refrigerator. 

### 2.4. Determination of Chlorogenic Acids

**Figure 1:**
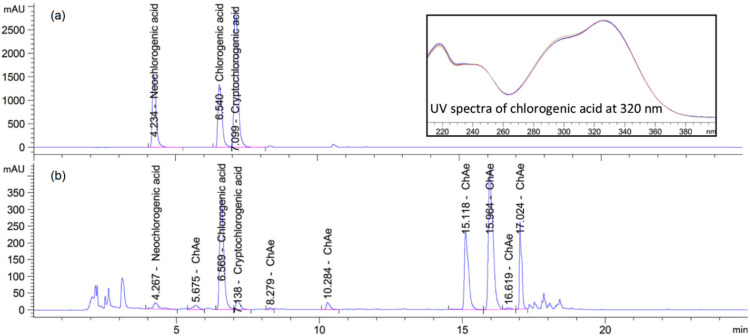
HPLC chromatogram of chlorogenic acids standards at 320 nm with UV spectrum (**a**) and chromatogram of the sweet potato sample of Beauregard variety after baking (**b**).

For HPLC analysis, prepared methanolic extracts were filtered through a Q-Max syringe filter (0.22 µm, 25 mm, PVDF) (Frisenette ApS, Knebel, Denmark). Chlorogenic acids were determined using Agilent 1260 Infinity HPLC (Agilent Technologies GmbH, Waldbronn, Germany) [29]. Using chromatographic analysis, the qualitative and quantitative data on the content of three monitored chlorogenic acids—chlorogenic (3-CQA), cryptochlorogenic (4-CQA), and neochlorogenic acid (5-CQA)—were collected. We also obtained qualitative data on the presence of substances, which, when comparing the UV spectrum at λ = 320 nm, had an agreement with chlorogenic acid ≥98.5%. We subsequently defined these substances as chlorogenic acid equivalents (CQAE), using chlorogenic acid calibration data to quantify them. These data are presented as ΣCQAE. For an illustrative example, Figure 1 shows a chromatographic record. Limits of detection for chlorogenic acid, neochlorogenic acid, and cryptochlorogenic acid were 0.22, 1.08, and 0.81 µg mL^−1^, respectively. Limits of quantification for chlorogenic acid, neochlorogenic acid, and cryptochlorogenic acid were 0.67, 3.36, and 2.54 µg mL^−1^, respectively.

### 2.5. Determination of the Total Polyphenol Content

The total polyphenol content (TPC) was determined using the colorimetric method by Lachman et al. [30]. Folin–Ciocalteu reagent was added to the aliquot volume of methanolic sample extract (0.1 mL) in the volumetric flask. After 3 min, 5 mL of sodium carbonate (20%) was used to provide an alkaline environment, and distilled water was added to the mark. Gallic acid was used as a standard to calculate the total polyphenol content. The absorbance of prepared solutions was measured after 2 h at the wavelength of 765 nm (spectrophotometer Shimadzu UV-1800, Kyoto, Japan). The results of the total polyphenol content in investigated samples were expressed as milligram gallic acid equivalents per gram of dry weight (mg GAE g^−1^ DW). All measurements were repeated four times, and the results were expressed as average ± SD for four replicates.

### 2.6. Determination of Antioxidant Activity

The antioxidant activity (AA) was determined by the method of DPPH radical scavenging activity and ferric reducing antioxidant power (FRAP) assay as described previously [31,32].

#### 2.6.1. DPPH Radical Scavenging Activity

The DPPH method is based on scavenging the stable free radical of 2,2′-diphenyl-1-picrylhydrazyl (DPPH). Trolox was used as a standard to calculate the antioxidant activity. A stock solution of DPPH was prepared by dissolving 0.025 g of DPPH in methanol (99.8%) and stored at 4 °C in the refrigerator. Before analysis, DPPH working solution was prepared from the DPPH stock solution by mixing with methanol (1:10). The analysis was performed as follows: an absorbance of DPPH working solution (*A*_0_) was measured at the wavelength of 515.6 nm by UV–VIS spectrophotometer (Shimadzu UV-1800, Kyoto, Japan). Subsequently, 0.1 mL of the sample extract was added to the cuvette with DPPH solution, evenly mixed, and left to stand for 10 min in darkness. After that, the absorbance (*A*_10_) was measured. The percentage value of DPPH inhibition was calculated based on the following equation:

$$DPPH\;inhibition(\%)=\left[(A_{0}-A_{10})/A_{0}\right]\times 100,$$

where *A*_0_ represents absorbance at time *t* = 0 (DPPH solution), and *A*_10_ is absorbance at time *t* = 10 min. 

The antioxidant activity was calculated and expressed as μmols of Trolox equivalents per gram of dry weight (μmol TE g^−1^ DW). All measurements were repeated four times, and the results were expressed as average ± SD for four replicates.

#### 2.6.2. Method of Ferric Reducing Antioxidant Power

The FRAP reagent was prepared as follows: a TPTZ solution (5 mmol L^−1^ in 40 mmol L^−1^ HCl), ferric chloride solution (10 mmol L^−1^), and acetate buffer (acetic acid, c = 0.1 mol L^−1^; sodium acetate, c = 0.1 mol L^−1^, pH 3.6) were mixed in a ratio of 1:1:10. Subsequently, 0.1 mL of sample extract was added to the FRAP reagent in the test tubes and evenly mixed (Heidolph Reax top, Heildolph Instruments GmbH, Schwabach, Germany). The absorbance at the wavelength of 593 nm was measured (spectrophotometer Shimadzu UV-1800, Kyoto, Japan) after 30-min incubation at 37 °C in the dark. Trolox was used as a standard.

The ferric reducing antioxidant power was calculated according to the calibration curve of Trolox and expressed as μmols of Trolox equivalents per gram of dry weight (μmol TE g^−1^ DW). All measurements were repeated four times, and the results were expressed as average ± SD (n = 4).

### 2.7. Statistical Analysis

The obtained data were expressed as mean ± standard deviation (SD) of quadruplicate (n = 4) experiments. After testing the dataset for normality (nonparametric distribution), the Kruskal–Wallis test and Wilcoxon test were used to determine the statistical differences using the RStudio (2020) software package [33]. Statistical differences between investigated varieties and among all heat treatment methods were reported at *p* < 0.05. The relationship between TPC, AA (DPPH and FRAP), and individual chlorogenic acids was determined by Spearman’s correlation coefficient.

## 3. Results and Discussion

### 3.1. Chlorogenic Acids

Chlorogenic acids are the main phenolic compounds in sweet potatoes. The major acid found in sweet potatoes is chlorogenic acid [21]. Chlorogenic acid (3-CQA) and isochlorogenic acid are the most effective scavengers of DPPH (2,2-diphenyl-1-picrylhydrazyl) free radicals and thus contribute to the antioxidant activity of sweet potatoes [34]. 

**Figure 2:**
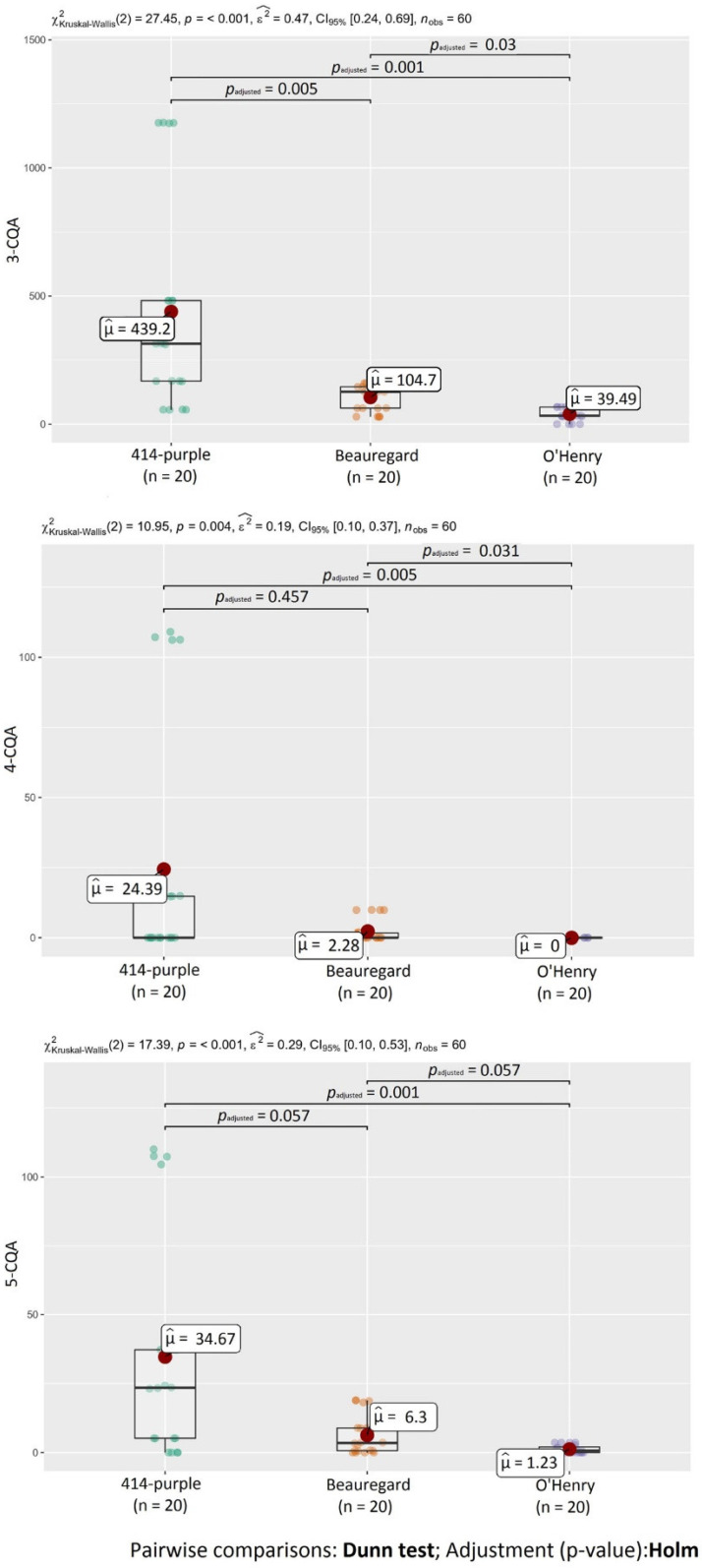
Statistical differences in the content of chlorogenic acids between monitored SP varieties.

**Figure 3:**
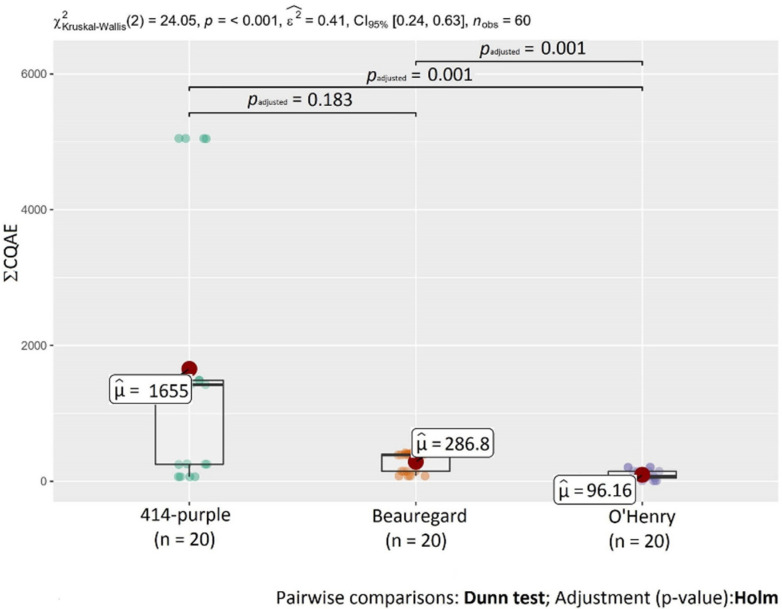
Statistical differences in the content of chlorogenic acid equivalents (ΣCQAE) between monitored SP varieties.

Table 1 shows the content of individual chlorogenic acids in non-treated and heat-treated sweet potatoes (SPs) of different flesh colors. Each variety showed different chlorogenic acid compositions. The most predominant chlorogenic acid in both non- and heat-treated SPs was 3-CQA followed by 5-CQA. The highest content of 3-CQA and 5-CQA was in the purple variety 414-purple (56.3 and 5.18 mg kg^−1^ DW, respectively). Steamed SPs of the 414-purple variety had the highest content of 4-CQA (107 mg kg^−1^ DW), followed by baked 414-purple SP (14.8 mg kg^−1^ DW). The chlorogenic acid content was variety-depended and decreased in the order of 414-purple > Beauregard > O’Henry. Generally, phenolic acids are more abundant in purple sweet potato varieties than white or orange varieties [35]. Differences between varieties may be related to genetic factors that play a significant role in secondary metabolites formation, including phenolic acids [36]. Statistical analysis confirmed the differences (*p* < 0.05) in the content of chlorogenic acids (Figure 2) between investigated SP varieties. On the other hand, there were no statistical differences in the content of 4-CQA, 5-CQA, and ΣCQAE between variety 414-purple and Beauregard and in the content of 5-CQA between varieties Beauregard and O’Henry (Figure 2 and Figure 3). However, even varieties with the same flesh color can differ in the total polyphenol content, antioxidant activity, and phenolic acid profile [11]. 

The presented results of chlorogenic acid contents are similar to the values measured in 10 SP varieties from China, with flesh color varying from white and yellow to dark purple. The content of 3-CQA in Chinese SP varieties with purple flesh ranged from <LOD to 53.4 mg kg^−1^ FW, and the 3-CQA content in orange-fleshed variety (XY34) was 13.7 mg kg^−1^ FW [37]. For comparison, in the varieties investigated in this study, the 3-CQA content was 27.8 and 8.06 mg kg^−1^ FW in 414-purple and Beauregard, respectively. The values of 3-CQA content in the raw sample of a variety Beauregard are comparable to those reported by [38] (28 mg kg^−1^ DW).

On the other hand, higher content of 3-CQA was reported in a Beauregard variety grown in the USA (205.5 mg kg^−1^ DW) [25], and in orange-fleshed (Beauregard-like) and white-fleshed SP from North Italy (436 and 221–333 mg kg^−1^ DW, respectively) [39]. Higher concentrations of chlorogenic acids were detected in four SP varieties (from white to purple-fleshed) from China (300–730 mg kg^−1^ DW of 3-CQA, 260–480 mg kg^−1^ DW of 5-CQA, and 600–930 mg kg^−1^ DW of 4-CQA) [40]. It can be assumed that not only variety but also the locality of cultivation significantly affects the content of chlorogenic acids in sweet potatoes.

Due to heat treatments, chlorogenic acid contents in sweet potatoes significantly increased. The content of 3-CQA ranged from <LOD (O’Henry—raw flesh) to 1175 mg kg^−1^ DW (414-purple—steamed), 4-CQA varied from <LOD to 107 kg^−1^ DW (414-purple—steamed), and 5-CQA ranged from ND to 107 mg kg^−1^ DW (414-purple—steamed). Considering all heat treatments used, steaming had the greatest effect on increasing the chlorogenic acid content, especially 3-CQA. In the 3-CQA content, statistical differences (*p* < 0.05) of content between non-treated SPs (raw flesh) and samples after microwaving, steaming, and baking were detected. In the Beauregard variety, baking resulted in the highest content of 4-CQA and 5-CQA (9.89 and 18.7 mg kg^−1^ DW, respectively). 

The significant increase of 3-CQA content was reported by [38] in four SP varieties (including Beauregard) grown in Egypt. In heat-treated samples (boiling, baking, microwaving, and deep-frying) of the Beauregard variety, the 3-CQA content ranged from 219 (microwaved) to 342 mg kg^−1^ DW (deep-fried). As a result of the heat treatment, the 3-CQA content was approximately 7.8, 7.9, 9.6, and 12.2 times higher in boiled, microwaved, baked, and deep-fried samples, respectively, than raw. Chlorogenic acid was also found as the most abundant phenolic acid in processed SP, followed by 3,5-dicaffeoylquinic acid [38]. Authors [41] also reported a positive effect of cooking methods on phenolic acid content in four sweet potato varieties. In the other study, the 3-CQA contents in steamed samples of varieties O’Henry, Beauregard, and 414-purple from Croatia were 1.89, 3.2, and 1.05 times higher, respectively, than in the raw sample [42].

However, some authors reported a significant decrease in phenolic acids due to heat treatments [24,35,43]. Regarding the 3-CQA content in SPs from North Italy, cooking methods (boiling, steaming, and microwaving) slightly reduced its concentration, while frying deeply reduced 3-CQA content compared to the raw sample [39].

Heating (100–200 °C) of 5-CQA water solution causes isomerization and transformation of chlorogenic acid. Dawidowicz et al. [44] reported fourteen compounds formed from 5-CQA due to heating of its water solution. The amount of each formed compound depends on the heating time and pH. 

**Figure 4:**
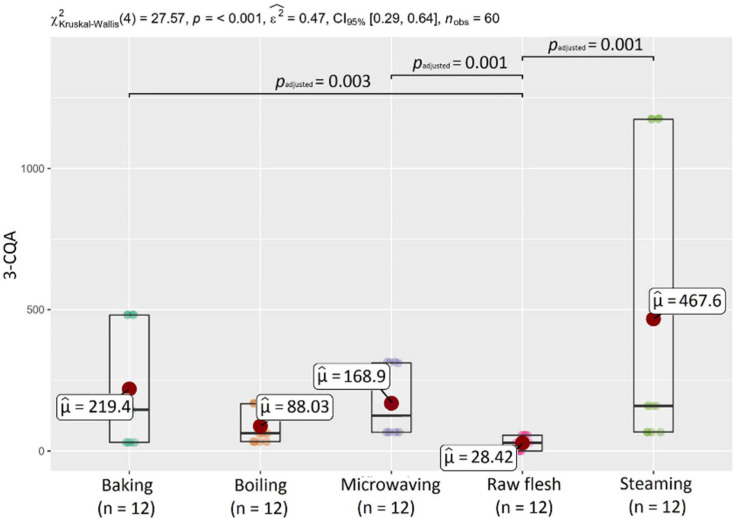

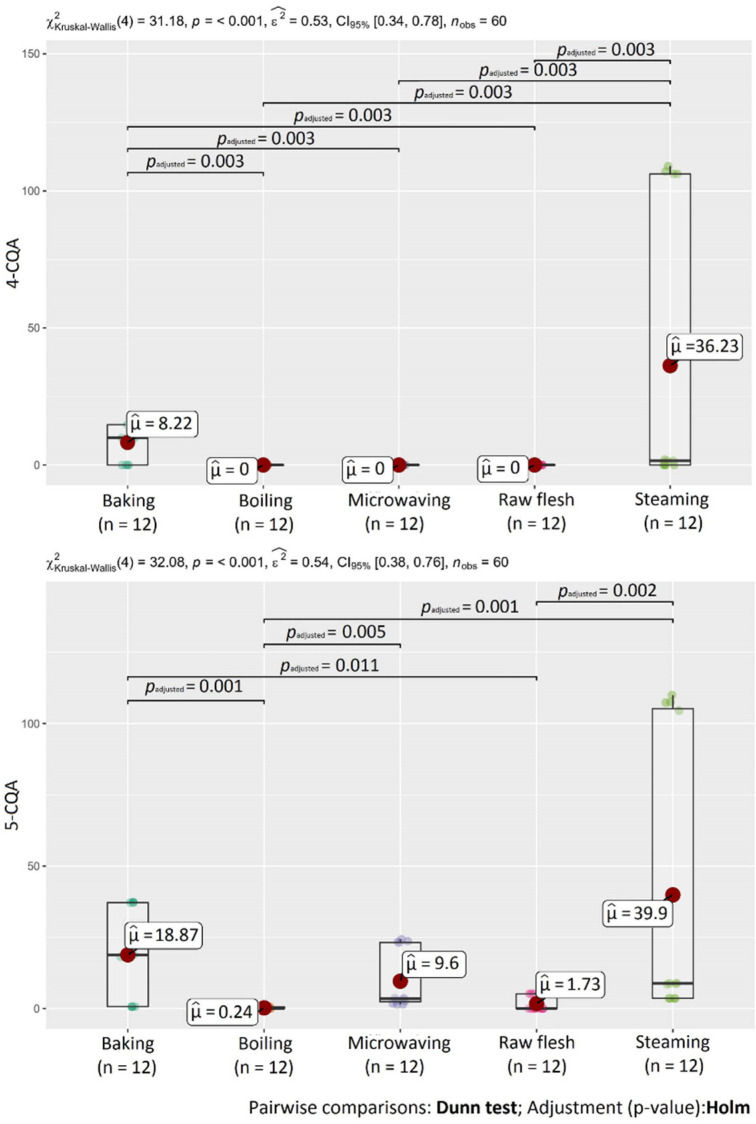
Statistical differences in chlorogenic acid contents between heat treatment methods.

**Figure 5:**
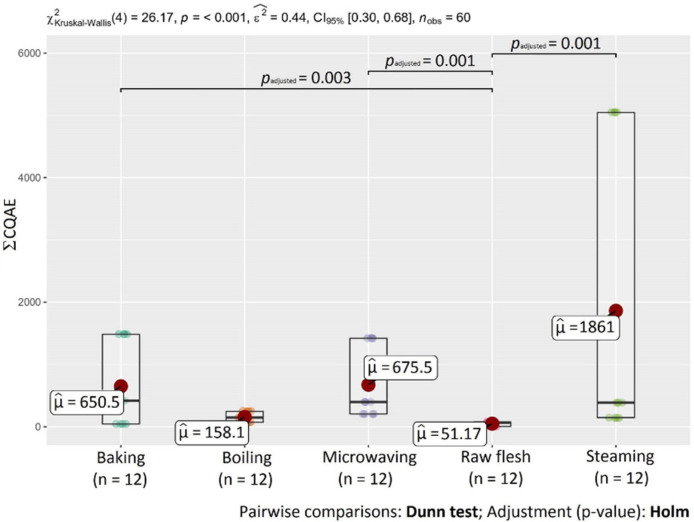
Statistical differences in the content of chlorogenic acid equivalents (ΣCQAE) between heat treatment methods.

Changes in phenolic acid content depend on the specific heating process [39]. Steaming had the greatest effect on the chlorogenic acid content; due to this heat treatment, the highest increase in the chlorogenic acid content in sweet potatoes was observed. This observation was also confirmed by statistical analysis. A statistically-significant difference (*p* < 0.05) in the content of chlorogenic acids was detected between raw flesh and steamed SPs (Figure 4). Figure 5 shows statistical differences in the content of ΣCQAE between raw and samples after baking, microwaving, and steaming. 

### 3.2. Total Polyphenol Content and Antioxidant Activity

**Figure 6:**
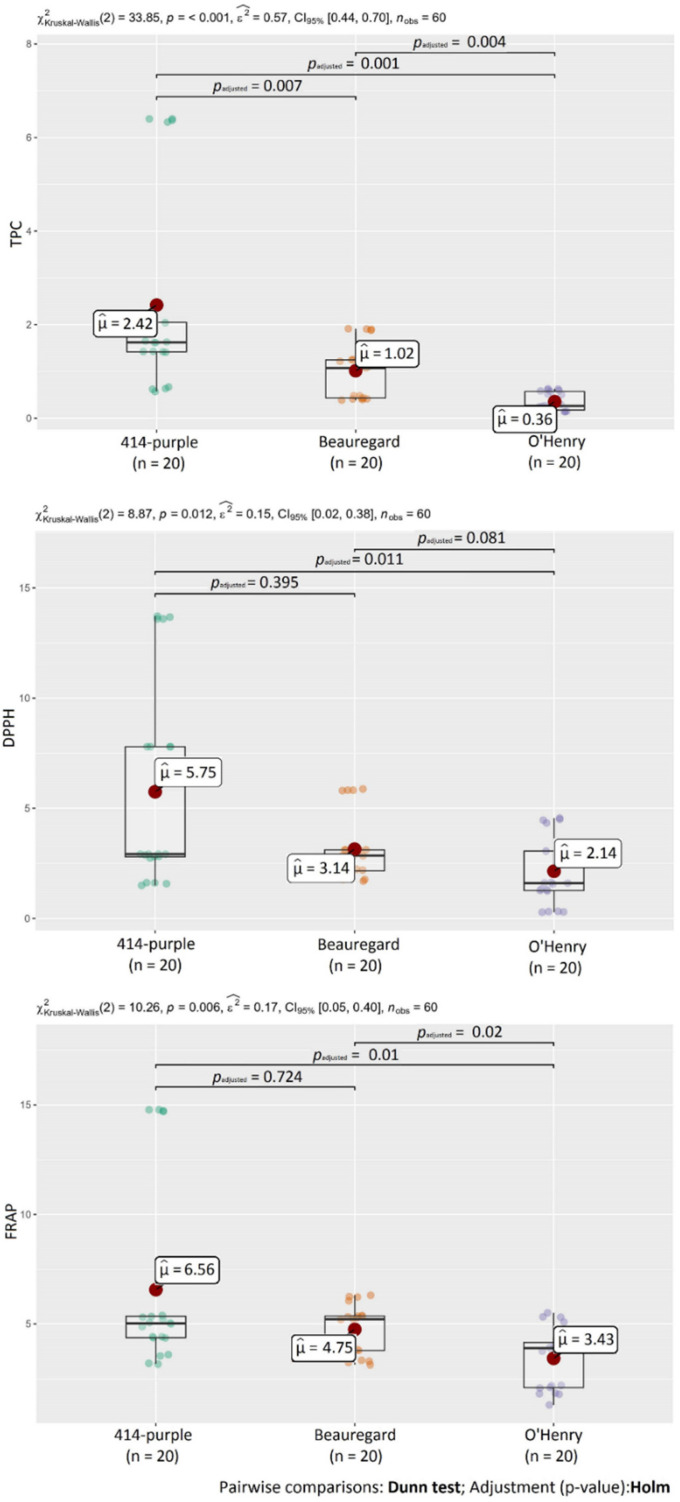
Statistical differences in TPC, DPPH, and FRAP between monitored SP varieties.

Total polyphenol content (TPC) was determined by a reference assay to measure polyphenols in the food using the Folin–Ciocalteu method. TPC values in non-treated SP ranged from 0.157 (O’Henry) to 0.623 mg GAE g^−1^ DW (414-purple). Based on these results, flesh color was a major factor influencing the polyphenol levels in sweet potatoes. This statement was also confirmed by the results, as the level of TPC in the purple variety (414-purple) was approximately 1.5 and 3.8 times higher than in the other two monitored varieties (Beauregard and O’Henry, respectively). Statistical differences were observed in TPC between all monitored SP varieties (Figure 6).

**Figure 7:**
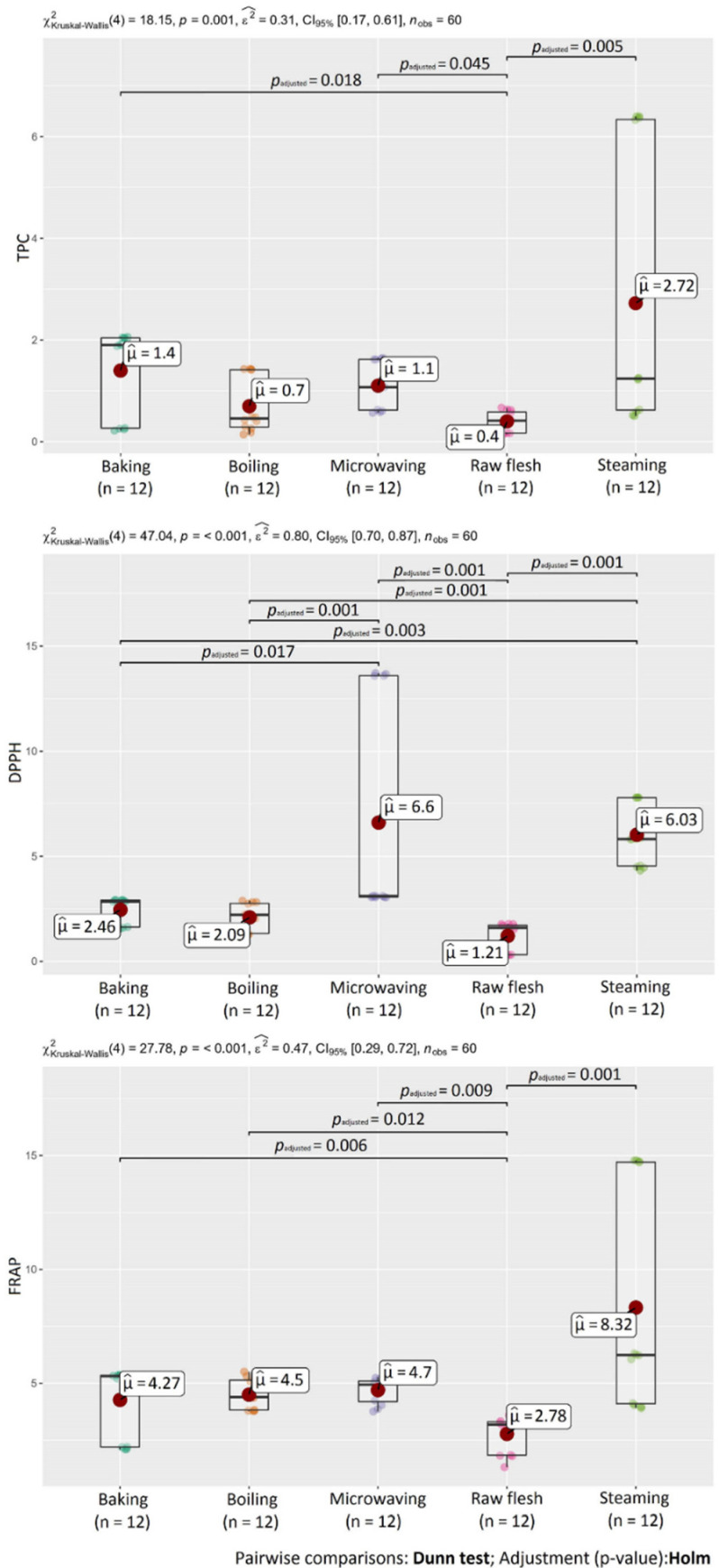
Statistical differences in TPC, DPPH, and FRAP between heat treatment methods.

Regarding the effect of heat processing, TPC was significantly influenced by the processing, which caused an increase of polyphenol content in sweet potatoes. Under the heat, some individual compounds are more easily released and react with the Folin–Ciocalteu reagent [35]. TPC values in heat-treated SP ranged from 0.218 (O’Henry—boiled) to 6.37 mg GAE g^−1^ DW (414-purple—steamed). Among the used methods, steaming had the greatest impact on polyphenols, which was also confirmed by statistical analysis (Figure 7). TPC in steamed samples was 10 times higher in variety 414-purple and about 3 times higher in varieties Beauregard and O’Henry compared to the raw sample. As described previously, a significant increase of TPC due to steaming may be the result of cell structure disruption, resulting in the simplified extraction of antioxidant compounds from the tissue [39]. 

Each heat treatment method had a different impact on the total polyphenol in monitored SP varieties. TPC in variety 414-purple decreased in order of steamed > baked > microwaved > boiled > non-treated, while in variety Beauregard, the order of TPC values was as follows: baked > steamed > microwaved > boiled > non-treated. Overall, the lowest TPC values were detected in non-treated samples of all monitored SP varieties. Considering heat treatment methods used, boiling caused only a slight increase of total polyphenols in all varieties.

Some previous studies also reported a positive effect of microwaving [38,39], steaming [39,42], baking [26,38], and boiling [26,38,39,45] on sweet potato polyphenols. Dincer et al. [26] attributed the increase in TPC to the release of phenolics by hydrolysis of glycoside bonds during heat processing and initiation of TPC oxidation through the catalytic activity of the polyphenol oxidase enzyme. 

However, [36,46] (in orange-fleshed) and [45] (in Beauregard) reported a significant decrease in TPC after thermal processing, and the loss of polyphenols could possibly also cause a reduction in AA.

To evaluate antioxidant activity (AA) of sweet potatoes, two different methods were used: 2,2′-diphenyl-1-picrylhydrazyl (DPPH) and ferric reducing antioxidant power (FRAP) assays. In non-treated sweet potatoes, AA ranged from 0.304 (O’Henry) to 1.76 μmol TE g^−1^ DW (Beauregard) for the DPPH method and from 1.70 (O’Henry) to 3.38 μmol TE g^−1^ DW (414-purple) for the FRAP assay. Overall, the purple variety presented the highest AA compared to the other two varieties with orange and white color, which was in agreement with previous studies [25,35,37,40,47,48,49,50]. In DPPH radical scavenging activity, a statistically significant difference (*p* < 0.05) was detected between variety 414-purple and O’Henry and in the FRAP assay between 414-purple and O’Henry and between Beauregard and O’Henry (Figure 6).

As in the case of TPC, antioxidant activity by the DPPH and FRAP assays increased as a result of the heat-processing in all investigated samples of SPs. AA ranged from 1.29 (O’Henry—boiled) to 13.6 μmol TE g^−1^ DW (414-purple—microwaved) for the DPPH method and from 2.15 (O’Henry—baked) to 14.8 μmol TE g^−1^ DW (414-purple—steamed) for the FRAP assay. Differences in values of AA could be the result of using two individual AA measuring methods. Many previous studies also reported a positive effect of different cooking methods on DPPH antioxidant activity [26,35,38,45,46,51]. In DPPH radical scavenging activity, statistical analysis confirmed differences between non-treated SPs (raw flesh) and samples after microwaving and steaming. In FRAP, statistical differences were detected between raw flesh and all heat treatment methods used in this study (Figure 7).

### 3.3. Correlations

**Figure 8:**
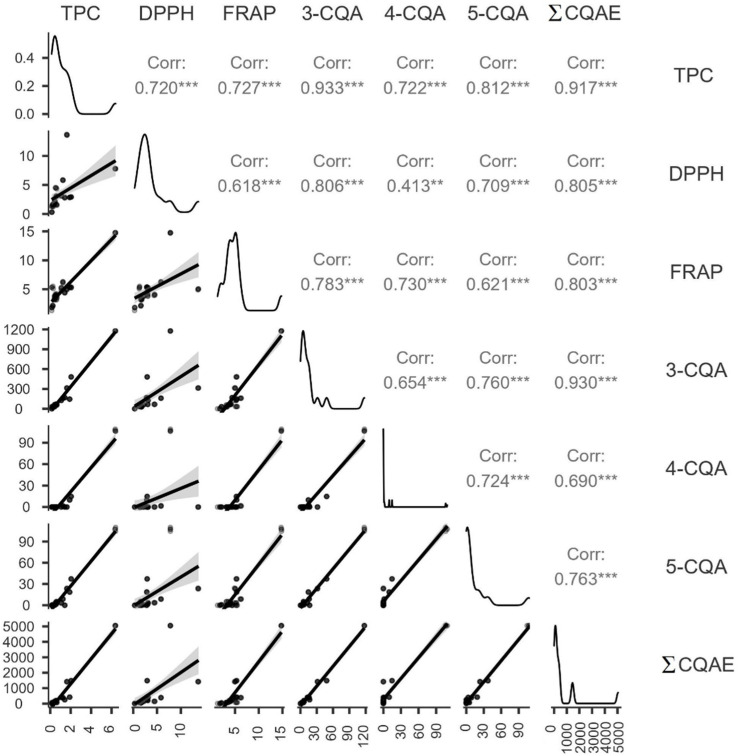
Spearman’s correlation coefficient between total polyphenol content (TPC), antioxidant activity (DPPH and FRAP), and individual chlorogenic acids in sweet potatoes (*** represents very strong correlation, ** represents strong correlation).

In addition to the measured data, Spearman’s correlation coefficient showed a relation between investigated parameters. 3-CQA, as the predominant chlorogenic acid in sweet potatoes, showed the strongest correlation with TPC. Figure 8 also shows a very strong (0.8–1.0) correlation between DPPH and 3-CQA, suggesting that 3-CQA is the most efficient DPPH free radical scavenger of the chlorogenic acids investigated in this study. Overall, a strong relationship was observed between the individual parameters monitored. 

## 4. Conclusions

In terms of the use and subsequent recommendation of sweet potatoes, not only for the food industry but also for consumers, further evaluation and research of different varieties of sweet potatoes is necessary to identify the variety with the most suitable content of bioactive compounds. The present study provides information on antioxidant activity and the content of selected bioactive compounds in sweet potatoes. In terms of AA, TPC, and chlorogenic acid contents, the 414-purple variety seems to be the most suitable for consumption. Based on AA and TPC, monitored varieties can be listed in decreasing order: 414-purple > Beauregard > O’Henry. The cooking techniques traditionally used in Slovakia, such as steaming, microwaving, baking, and boiling, caused an increase of antioxidant activity and phenolic content in all monitored varieties. It can be assumed that heat treatment has a positive effect on the total polyphenol content, antioxidant activity, and chlorogenic acids content in sweet potatoes. However, the impact of individual heat treatments on the monitored parameters—AA, TPC—and the content of selected chlorogenic acids cannot be unambiguously defined because each variety reacts differently to the heat treatments used. 

The presented results can bring a more comprehensive understanding of the processing impact on bioactive compounds in sweet potatoes and thus contribute to the improvement of product processing technology. Information provided by our study can also enhance consumer awareness and thus increase the consumption of sweet potatoes in Slovakia.

## Figures and Tables

**Table 1:** Individual chlorogenic acids, total polyphenol content, and antioxidant activity in raw and heat-treated sweet potatoes.

Treatment	Variety	Chlorogenic Acids mg kg^−1^ DW				TPCmg GAE g^−1^ DW	Antioxidant Activity μmol TE g^−1^ DW	
		3-CQA	4-CQA	5-CQA	ΣCQAE		DPPH	FRAP
Non-treated	Beauregard	29.0 ± 0.18	<LOD	<LOD	79.2 ± 0.58	0.413 ± 0.02	1.76 ± 0.03	3.25 ± 0.07
	O’Henry	<LOD	<LOD	<LOD	7.05 ± 0.07	0.157 ± 0.01	0.304 ± 0.02	1.70 ± 0.22
	414-purple	56.3 ± 0.66	<LOD	5.18 ± 0.02	67.2 ± 0.35	0.623 ± 0.04	1.58 ± 0.05	3.38 ± 0.02
Microwaved	Beauregard	126 ± 0.09	<LOD	3.34 ± 0.51	399 ± 0.88	1.08 ± 0.02	3.11 ± 0.00	5.12 ± 0.20
	O’Henry	66.6 ± 0.03	<LOD	1.86 ± 0.67	206 ± 0.59	0.595 ± 0.02	3.06 ± 0.01	3.98 ± 0.18
	414-purple	313 ± 2.28	<LOD	23.6 ± 0.63	1421 ± 2.11	1.63 ± 0.01	13.6 ± 0.05	5.00 ± 0.07
Steamed	Beauregard	159 ± 0.37	1.52 ± 0.37	8.77 ± 0.21	388 ± 0.76	1.24 ± 0.01	5.83 ± 0.03	6.21 ± 0.09
	O’Henry	67.1 ± 0.10	<LOD	3.59 ± 0.02	146 ± 1.59	0.562 ± 0.05	4.46 ± 0.08	4.01 ± 0.08
	414-purple	1175 ± 1.51	107 ± 1.35	107 ± 2.75	5048 ± 1.74	6.37 ± 0.03	7.79 ± 0.00	14.8 ± 0.03
Baked	Beauregard	145 ± 0.24	9.89 ± 0.02	18.7 ± 0.44	418 ± 0.18	1.90 ± 0.01	2.85 ± 0.00	5.35 ± 0.02
	O’Henry	30.6 ± 0.14	<LOD	0.70 ± 0.01	47.0 ± 0.20	0.246 ± 0.02	1.60 ± 0.03	2.15 ± 0.05
	414-purple	481 ± 0.86	14.8 ± 0.08	37.2 ± 0.06	1485 ± 2.27	2.05 ± 0.01	2.92 ± 0.00	5.31 ± 0.07
Boiled	Beauregard	62.7 ± 0.29	<LOD	0.717 ± 0.10	149 ± 0.22	0.448 ± 0.04	2.18 ± 0.08	3.80 ± 0.02
	O’Henry	33.2 ± 0.09	<LOD	<LOD	73.6 ± 0.08	0.218 ± 0.06	1.29 ± 0.03	5.30 ± 0.15
	414-purple	168 ± 0.99	<LOD	<LOD	251 ± 3.96	1.42 ± 0.01	2.81 ± 0.05	4.39 ± 0.02

The values are expressed as mean ± SD. 3-CQA: chlorogenic acid; 4-CQA: cryptochlorogenic acid; 5-CQA: neochlorogenic acid; ΣCQAE: chlorogenic acid equivalents; TPC: total polyphenol content; GAE: gallic acid equivalent; DW: dry weight; DPPH: 2,2′-diphenyl-1-picrylhydrazyl; FRAP: ferric reducing antioxidant power; TE: Trolox equivalent; <LOD: below limit of detection.

## Data Availability

The datasets generated for this study are available upon request to the corresponding author.
